# Neurogenic and neuro-protective potential of a novel subpopulation of peripheral blood-derived CD133+ ABCG2+CXCR4+ mesenchymal stem cells: development of autologous cell-based therapeutics for traumatic brain injury

**DOI:** 10.1186/scrt151

**Published:** 2013-01-06

**Authors:** Joan E Nichols, Jean A Niles, Douglas DeWitt, Donald Prough, Margaret Parsley, Stephanie Vega, Andrea Cantu, Eric Lee, Joaquin Cortiella

**Affiliations:** 1 Laboratory of Tissue Engineering and Regenerative Medicine, 301 University Boulevard, Mail Route, 0435, University of Texas Medical Branch, Galveston, Texas, 77555-0435, USA; 2 Departmen. of Internal Medicine, Division of Infectious Diseases, 301 University Boulevard, Mail Route, 0435, University of Texas Medical Branch, Galveston, Texas, 77555-0435, USA; 3 Department of Anesthesiology, 301 University Boulevard, Mail Route, 0591, University of Texas Medical Branch, Galveston, Texas, 77555-0591, USA

## Abstract

**Introduction:**

Nervous system injuries comprise a diverse group of disorders that include traumatic brain injury (TBI). The potential of mesenchymal stem cells (MSCs) to differentiate into neural cell types has aroused hope for the possible development of autologous therapies for central nervous system injury.

**Methods:**

In this study we isolated and characterized a human peripheral blood derived (HPBD) MSC population which we examined for neural lineage potential and ability to migrate *in vitro *and *in vivo*. HPBD CD133+, ATP-binding cassette sub-family G member 2 (ABCG2)+, C-X-C chemokine receptor type 4 (CXCR4)+ MSCs were differentiated after priming with β-mercaptoethanol (β-ME) combined with *trans*-retinoic acid (RA) and culture in neural basal media containing basic fibroblast growth factor (FGF2) and epidermal growth factor (EGF) or co-culture with neuronal cell lines. Differentiation efficiencies *in vitro *were determined using flow cytometry or fluorescent microscopy of cytospins made of FACS sorted positive cells after staining for markers of immature or mature neuronal lineages. RA-primed CD133+ABCG2+CXCR4+ human MSCs were transplanted into the lateral ventricle of male Sprague-Dawley rats, 24 hours after sham or traumatic brain injury (TBI). All animals were evaluated for spatial memory performance using the Morris Water Maze (MWM) Test. Histological examination of sham or TBI brains was done to evaluate MSC survival, migration and differentiation into neural lineages. We also examined induction of apoptosis at the injury site and production of MSC neuroprotective factors.

**Results:**

CD133+ABCG2+CXCR4+ MSCs consistently expressed markers of neural lineage induction and were positive for nestin, microtubule associated protein-1β (MAP-1β), tyrosine hydroxylase (TH), neuron specific nuclear protein (NEUN) or type III beta-tubulin (Tuj1). Animals in the primed MSC treatment group exhibited MWM latency results similar to the uninjured (sham) group with both groups showing improvements in latency. Histological examination of brains of these animals showed that in uninjured animals the majority of MSCs were found in the lateral ventricle, the site of transplantation, while in TBI rats MSCs were consistently found in locations near the injury site. We found that levels of apoptosis were less in MSC treated rats and that MSCs could be shown to produce neurotropic factors as early as 2 days following transplantation of cells. In TBI rats, at 1 and 3 months post transplantation cells were generated which expressed markers of neural lineages including immature as well as mature neurons.

**Conclusions:**

These results suggest that PBD CD133+ABCG2+CXCR4+ MSCs have the potential for development as an autologous treatment for TBI and neurodegenerative disorders and that MSC derived cell products produced immediately after transplantation may aid in reducing the immediate cognitive defects of TBI.

## Introduction

Studies examining repair mechanisms in the brain have shown that neural precursor cells have the capacity to migrate to injury sites and differentiate into neurons [1-4]. Unfortunately, neurogenesis from endogenous stem cells is not sufficient to produce meaningful levels of recovery after injury [4,5]. Augmentation of endogenous stem cells with cells from sources other than the brain may enhance neurogenesis sufficiently to promote meaningful recovery. Mesenchymal stem cells (MSC) have already been shown to provide therapeutic benefits in animal models for a variety of neurological disorders including stroke, Parkinson's disease and traumatic brain injury (TBI) [6,7]. The potential of MSC to differentiate into neural cell types has aroused hope for the possible development of autologous therapies for central nervous system (CNS) injury for both injured civilian and active duty military personnel [8]. Current data have also suggested that MSC may also provide a source for supportive factors that aid immune modulation or neuro-protection, aiding in recovery [9]. Although MSC have great potential, standard conditions for isolation based on definitive sets of cell markers that relate to efficiency of MSC to consistently develop into neural lineages, have yet to be established. Thus, the inconsistent results of some studies using MSC to treat neurological conditions may be due in part to variations in culture conditions, long-term passage of cells, or to the use of mixed populations of MSC at slightly different developmental stages. Although few neuron-specific markers have been identified, early stem cell markers have been described that are indicative of immature cell status. Such markers include expression of CD133 [10,11], either alone [12], or in combination with stage-specific embryonic antigen-4 (SSEA-4) [13,14] or ATP-binding cassette sub-family G member 2 (ABCG2) [15]. Many of these markers have been used to positively select for neural stem cells from fetal brain or other tissue sources [12-17], yet few studies have focused on neural differentiation of isolated MSC from non-mobilized peripheral blood (PB) [18,19].

In this study we isolated and characterized a human PB-derived (HPBD) MSC population, which we examined for neural lineage potential and ability to migrate *in vitro *and *in vivo*. We focused our attention on identifying MSC subpopulations based on co-expression of the immature stem cell markers CD133, SSEA-4, ABCG2 and chemokine receptor type 4 (CXCR4), a molecule that has been shown to be involved in stromal derived factor-1 (SDF-1)/CXCR4-mediated migration and plays an important role in the regulation of stem/progenitor cell trafficking [20-23]. After sorting subpopulations based on expression of immature stem cell markers we examined each sorted population for their capability of neural marker induction following trans-retinoic acid (RA) priming. We identified a population of HPBD CD133+ABCG2+CXCR4+ MSC with high neuronal marker induction efficiency. Transplantation of RA-primed CD133+ABCG2+CXCR4+ MSC into the lateral ventricle of uninjured rats, or those subjected to parasagittal fluid percussion TBI, resulted in survival of cells, migration to the injury site, improvement in cognitive functioning of TBI animals, differentiation of cells and reduction in induction of apoptosis at the injury site after transplantation. Transplanted MSC were shown to produce the neurotropic factors chondroitin sulfate proteoglycan (CSPG) and brain derived neurotropic factor (BDNF). The ability to obtain MSCs from an accessible source such as PB makes them an excellent candidate for use as an autologous stem cell therapy for the treatment of TBI and neurodegenerative disorders.

## Materials and methods

### Isolation of MSC

PB buffy coats were obtained from the Gulf Coast Blood Bank or from volunteer donors (18 to 50 years of age). PB was drawn from ten donors with informed consent using University of Texas Institutional Review board approved protocols. The mononuclear cell (MNC) fraction was isolated using Ficoll density gradient separation medium (GE Health Care-Biosciences, Pittsburg, PA, USA). Subpopulations of MNC were isolated by counter-current centrifugal elutriation using a Beckmann J6M elutriator (Beckman-Coulter Instruments, Brea, CA USA) in a Sanderson chamber. A Masterplex peristaltic pump (Cole Parmer Instruments, Vernon Hills, IL, USA) was used to provide the counter-current flow. RPMI 1640 supplemented with 2 mM glutamine, 100 units penicillin G and 100 μg/ml streptomycin, and 10% donor-derived autologous serum was used as elutriation medium. We loaded 3 to 6 × 10^6 ^cells at 3000 RPM and elutriation fractions were isolated using a stepwise reduction of rotor speed and media flow to allow for collection of subpopulations of MNC based on size and density. A 4- to 6-μm cell fraction was collected and the phenotype of this cell population was determined.

Cells were plated in 75- or 150-cm^2 ^tissue culture flasks in DMEM (Sigma, St Louis, MO, USA), 0.1 mM nonessential amino acids, 100-U/ml penicillin and streptomycin, and 1 ng/ml basic fibroblast growth factor (FGF) with 10% fetal calf serum, plus 0.2 mM L-glutamine. The majority of cells were found to be strongly adherent to plastic after 8 to 24 hours of culture.

### Characterization of MSC

Antibodies for phenotyping conjugated to fluorescein isothiocyanate (FITC), phycoerytherin (PE) or PerCP were purchased from companies listed in Table 1 and were used as described by each manufacturer. Corresponding immunoglobulin (IgG)-matched isotype control antibodies were used to set baseline values for analysis markers. Staining for ABCG2 (Stem Cell Technologies, Vancouver, Canada), CD133 (Miltenyi Biotech, Auburn, CA, USA), CD105, CD90, CD29, and CXCR4 (BD Biosciences, San Jose, CA, USA) was done with PE conjugated antibodies. Elutriated MSC were stained in PBS (Ca- and Mg-free) supplemented with 5% autologous serum. After the final wash, cells were kept at 4°C in neurobasal media prior to culture, or were fixed with 2% paraformaldehyde (PF) before analysis using a FACSAria instrument (BD Biosciences,), with acquisition and analysis using the FACSDiva program (BD Biosciences). Isolated cells expressed MSC markers CD105, CD90, CD29 by flow cytometry and were negative for expression of markers of hematopoietic lineage, CD14, CD34, CD45 and Lineage-1 (Lin-1) as has been previously described for bone marrow MSC [19].

**Table 1:** Classification of the 4 to 6 micron elutriated mononuclear cell fraction

Cell lineage marker	Reagent Source	Surface antigen	RFI pre-priming, mean +/- SD (% positive)	RFI Post priming, mean +/- SD (% positive)
**Leukocyte**	BD	CD45 (pan leukocyte)	6.3 +/- 1.1 (0%)	5.4 +/- 0.7 (0%)
	BD	CD14 (macrophage)	8.3 +/- 2.1 (0%)	6.2 +/- 1.4 (0%)
	BD	CD3 (T Cell)	5 +/- 0.2 (0%)	6.1+/- 1.6 (0%)
	BD	T cell receptor (T Cell)	6.3 +/- 1.4 (0%)	4.5 +/- 1.1 (0%)
	BD	CD19 (B cell)	6.8 +/- 0.7 (0%)	7.4 +/- 1.6 (0%)
	BD	Immunoglobulin (B Cell)	7.9 +/- 2.3 (0%)	7.6 +/- 3.1 (0%)
	BD	CD13 (myeloid lineage)	8 +/- 2.4 (0%)	5.5 +/- 2.1 (0%)
**Endothelium**	BD	CD31	5.9 +/- 2.2 (0%)	6.1 +/- 3.1 (0%)
**Fibroblast**	BD	CD104a	4.7 +/- 1.1 (0%)	5.1 +/- 2.4 (0%)
	BD	CD104b	6.2 +/- 2.4 (0%)	7.3 +/- 3.2 (0%)
**Chemokine**	BD	CXCR4	1236.2 +/- 362.2 (92.1 +/5.8%)	1422.1 +/- 303.2 (86.1 +/- 8.3%)
	BD	CXCR3	7.2 +/- 3.3 (0%)	6.2 +/- 2.7 (0%)
	BD	CCR5	8.2 +/- 3.2 (0%)	7.1 +/- 2.7 (0%)
**Stem cell**	BD, CH	SSEA-1	93 +/- 5.5 (35.2 +/- 5.2%)	5.4 2 +/-0.5 (5.4 +/- 1.6%)**
	BD, CH	SSEA-4	206.7 +/63 (41.5+/-6.9%)	5.39 +/- 0.7(0%)**
	BD	CD34	6.1 +/- 2.8 (0%)	7.2 +/- 3.4 (0%)
	SCT	ABCG2	670.9 +/- 38.7 (84.9 +/- 5.4%)	127.3 +/- 35.4 (31.5 +/- 5.7%)**
	MIL, BD	CD133	688.8 +/- 63 (95.1+/- 2%)	127.8 +/- 7.4 (39.2 +/- 7.8%)**
	BD	CD105	1892 +/- 49 (93& +/- 22)	855 +/- 34 (86 +/- 14%)*
	BD	CD90	1568 +/- 42 (96% +/- 54)	544 +/- 22 (74 +/-11)**
	BD	CD29	446 +/- 22 (85% +/-16%)	349 +/- 56 (61 +/- 22%)
**Human Leukocyte Antigens**	BD	MHC Class-1 (human)	38.6 +/- 8 (35+/- 6.2%)	59.5 +/-10 (59.4+/- 7.2%)
	BD	MHC Class II	11.3 +/- 3.79 (0%)	12.2 +/- 3.7 (0%)
**Neuronal Markers**	CH	Nestin	11.3 +/- 3.9 (0%)	143.7 +/- 42 (23.1 +/- 4.4%)*
	CH	Map-1B	11.3 +/- 3.9 (0%)	139.3 +/- 44. 2(16+/- 2.9%)*
	CH, DAKO	GFAP	4.2 +/- 1.6 (0%)	7.4 +/- 1.7 (0%)
	CH	Tuj1	2.6 +/- 1.1 (0%)	18.5 +/- 2.4 (3.4+/- 1.6%)
	CH, CST	TH	2.7 +/- 0.8(0%)	6.2 +/- 2.4 (2.1+/- 0.5%)

Immunocytochemistry was used to examine expression of hematopoietic-, fibroblast-, embryonic- or progenitor cell-associated cluster of differentiation (CD) antigens or markers indicative of neural lineage selection. Staining was evaluated by quantitative flow cytometry using well-defined antibodies. This table shows the occurrence of positive cells (% of cells positive) and relative fluorescent intensity (RFI) of freshly isolated cells before and after RA priming. Statistically significant comparisons for markers include those that decreased (*P < 0.0.001) and increased (**P < 0.05) between isolation and 24 hours post priming. MHC, major histocompatibility complex; CXCR4, CXC chemokine Receptor-4; MAP-1β, microtubule associated protein-1; Tuj1, neuron specific class III β-tubulin; TH, tyrosine hydroxylase; SSEA-4, stage specific embryonic antigen; GFAP, glial fibrillary acidic protein. Reagent sources Becton Dickenson, BD; Chemicon, CH; Stem Cell Technologies, SCT; Cell Signaling Technologies, CST; Miltenyi, MIL.

Phagocytic capacity of elutriated cells was evaluated using uptake of fluorescent beads as described previously [24]. Selected elutriation fractions of cells were exposed to 3.5 μg/ml lipopolysaccharide (LPS) (Sigma Aldrich), or 4 ug/ml phytohemagglutinin-M (PHAM), and supernatants were evaluated for cytokine production. The BD Biosciences TH1/TH2 flow cyometric cytokine bead array kit was used to measure IL-2, IL-4, IL-5, IL-10, TNF and IFN-γ protein levels produced by elutriated cells pre- and post RA priming as well as post exposure to LPS or PHAM as described by the manufacturer.

### Induction of neuronal markers

Mononuclear cells (MNC) isolated from 10 blood donors were subjected to counter-current centrifugal elutriation and a 4- to 6-μm cell fraction was collected by elutriation followed by fluorescence activated cell sorting (FACS) based on co-expression of CXCR4 and CD133, CD133 and ABCG2, or CD133 and ABCG2 and SSEA-4. MSC were primed for induction of neural lineage markers by culture in DMEM-LG supplemented with 10% human AB serum, 10^-3 ^M β-mercaptoethanol (β-ME) (Sigma); 5 × 10^-7 ^M all-*trans*-retinoic acid (RA) (Sigma) for 24 hours [25-28]. Viability of cells was determined using a Molecular Probes LIVE/DEAD Viability/Cytotoxicity Kit (Invitrogen, San Jose, CA, USA). Cells were then cultured in neural differentiation media (NDM) which was composed of neurobasal medium (Invitrogen, Carlsbad, CA, USA) with addition of 2 mM L-glutamine, B-27 supplement (Invitrogen), 25 ng/ml FGF2, 10 ng/ml epidermal growth factor (EGF), and 10% autologous or human AB serum.

### Immunocytochemistry

Neural marker expression was examined using immunostaining followed with evaluation by flow cytometry and/or fluorescent microscopy. Neural markers that were used to evaluate potential for neural marker expression for selected subpopulations of the MSC included microtubule associated protein-1 β (MAP-1β), nestin, tyrosine hydroxylase (TH), type III beta-tubulin (Tuj1), neuronal specific nuclear protein (NEUN), glial fibrillary acidic protein **(**GFAP**)**, choline acetyltransferase (CHAT) enzyme and galactocerebroside (GalC) using antibodies described below. Nestin was chosen because it is expressed during neuronal development but is also expressed in non-neural stem cell populations. MAP-1β, although not restricted in expression to neuronal cells, is present at high levels in the embryonic and newborn rat brain and is an early neuronal microtubule-associated protein that is involved in microtubule assembly. TH is a 56 to 60 kD structural protein that is expressed in the neurons of the peripheral nervous system (PNS) and CNS, but is also expressed by non-neuronal tissues. Tuj1 contributes to microtubule stability in neuronal cell bodies and axons, and plays a role in axonal transport. Antibodies against NEUN specifically recognize the DNA-binding, neuron-specific protein, NEUN, which is present in most CNS and PNS neuronal cell types of most vertebrates. GFAP is an intermediate filament protein that is thought to be specific for astrocytes in the CNS, and expression of this protein distinguishes astrocytes from other glial cells. The neurotransmitter, acetylcholine, is synthesized from choline and acetyl-coA by the CHAT enzyme, making CHAT a specific marker of cholinergic neurons. The regulation of CHAT activity is thought to be important during the development of nervous systems interactions. GalC is a specific cell-surface antigenic marker for oligodendrocytes and Schwann cells. GalC is also a major galactosphingolipid of myelin, which plays a role in myelination.

Preparations for imaging were mounted in Slow Fade GOLD with 4',6-diamidino-2-phenylindole (DAPI) (Molecular Probes, Eugene, OR, USA) and observed using an LSM 510 Meta advanced laser scanning confocal microscope (Zeiss, Thornwood, NY, USA). Fluorescent microscopy was done using a Zeiss Axioscope Fluorescent microscope or a Nikon T300 Inverted Fluorescent microscope (Nikon Inc, Melville, NY, USA.). For brain sections, all positive cells on a slide were counted. Three replicate evaluations of cell counts for stained slides were performed and averaged, and then counts were later checked by a second observer.

In a subset of experiments cells were stained to identify expression of neuroprotective products after 7 days of cell culture. RA-primed or unprimed cells were stained for expression of chondroitin sulfate proteoglycan (CSPG), and brain derived neurotropic factor BDNF. For expression of CPSG by *in vitro *cultured MSCs, Alexa Fluor Anti-chondroitin sulfate (BD Pharmingen) was used. For identification of CSPG in brain sections anti-CPSG unlabeled human specific antibody (EMD Millipore, MA, USA) was used as described by the manufacturer. For identification of expression of BDNF in cultured cells or brain sections Anti-human Pro-BDNF (R & D Systems, Minneapolis, Billerica, MN, USA) was used as described by the manufacturer. Secondary antibodies used for staining brain sections were conjugated to anti-mouse rhodamine.

### Flow cytometry

For flow cytometry, cells were fixed with 2% (w/v) PF for 30 minutes at 37°C, washed in PBS and then permeabilized in 1% BD permeabilizing solution (BD Biosciences) for 10 minutes with a final wash in tris buffered saline (TBS). Nonspecific binding was blocked by a 1-hour treatment in TBS plus 0.1% w/v Tween containing defatted milk powder (30 mg ml^-1^). Cells were incubated for 1 hour at 37°C with one of the following primary antibodies (diluted in blocking buffer): nestin (EMD Milipore, 1:200 dilution), CHAT (EMD Milipore, 1:250 dilution), GalC (EMD Milipore, 1:200 dilution), GFAP (Dako, Glostrup, Denmark, 1:500 dilution), TH (Cell Signaling Technologies, Danvers, MA, USA and EMD Milipore, 1:200 dilution), Tuj1 (Eurogentec, Southampton, Hampshire, UK, 1:1,500 dilution), MAP-1 β (EMD Milipore, 1:200 dilution) and SSEA-4 (EMD Milipore, 1:300 dilution). After three washes in TBS, cells were incubated in secondary antibodies conjugated to FITC, rhodamine, or Cy5 anti-mouse, anti-rat, or anti-rabbit IgGs (1:500 dilution) for 1 hour at 37°C, washed then stored at 4°C until analyzed. Use of isotype-matched controls and omission of primary antibodies served as negative controls and resulted in less than 2% background staining for flow cytometry analysis of samples.

Positive cells identified by expression of each marker, or set of markers, were sorted using flow cytometry, and cytospins of positively sorted cells were made and examined using fluorescent and confocal microscopy as described above. To validate our immunostaining for flow cytometric analysis we examined location of staining or presence of appropriate staining patterns (intracellular, nuclear or extracellular) of the cells on the cytospin slides. For some phenotype evaluations we compared manual counts of cytospin slides to the flow cytometric data to make sure that we were analyzing whole cells and not cell debris or damaged cells.

### Immunoprecipitation

TH and nestin production was also shown by immunoprecipitation of these proteins followed by gel electrophoresis. CD133+ABCG2+CXCR4+ cells were incubated for 6 hours in 0.5 mCi of ^35^S-methionine (GE Healthcare Biosciences)in 2 ml of methionine-free DMEM (MP Biomedicals, Irvine CA, USA) and then cells were lysed using 1% NP-40 in 50 mM Tris-HCL with 150 mM NaCl, pH 8, buffer. For immunoprecipitation of newly produced neuronal proteins 10 μg of antibody was added to 50 μl of ^35^S methionine labeled and pre-cleared lysate and was incubated at 4°C for 1 hour [29]. After washing, 50 μl of protein A slurry in pre-chilled lysis buffer was added and the sample was incubated for 1 hour at 4°C. After the last wash, the sample supernatant was aspirated and 50 μl of 1× Laemmli sample buffer was added to each bead pellet. Samples were vortexed and heated to 90 to 100°C for 10 minutes. Samples and MW standards with known concentrations were separated by electrophoresis. Autoradiography was used to evaluate protein bands.

### Evaluation of micro-environmental cues on *in vitro *induction of neuronal markers

1 × 10^5 ^unprimed or RA-primed CD133+ABCG2+CXCR4+ MSC were FACS-sorted and placed in the bottom of a transwell plate with a 0.2 um filter; 1 × 10^6 ^cells from four different neuronal cell lines or from a bone marrow stromal cell line (lines purchased from American Type Culture Collection, ATCC, Manassas, VA, USA) were placed in the top chamber. Cell lines used included human astroglial cell line (SVG p12), rat astrocyte line (DITNC 1), human neuroblastoma line (SK-N-FI), human dopaminergic line (SK-N-MC), and a human bone marrow stromal cell line (HS-5). After 7 days of culture, the CD133+ABCG2+CXCR4+ MSC efficiency of neuronal lineage differentiation was evaluated by staining for expression of nestin, GFAP, CHAT, TH, Tuj1 or GALC with analysis by FACS. The cells that were positive for expression of nestin, GFAP, CHAT, TH, Tuj1, or GALC were sorted and examined using fluorescent microscopy. Cytospin preparations were performed as described by the manufacturer (Shandon, Pittsburg, PA, USA). In a subset of experiments (*n *= 8) in order to evaluate the antibodies chosen for neuronal marker analysis, MSC were cultured for 7 days, cytospins of cells were made, slides were fixed in 2% freshly made PF and were then stained for expression of neuronal markers.

To observe the influence of direct cell-to-cell interactions on differentiation, we placed 1 × 10^5 ^unprimed or RA-primed CD133+ABCG2+CXCR4+ MSC in co-culture with 1 × 10^6 ^carboxyfluorescein diacetate sucinimydyl ester (CFSE)-labeled cell lines listed above following manufacturer's instructions (Molecular Probes,). After 7 days of culture, efficiency of neuronal lineage differentiation was evaluated with analysis using flow cytometry by gating on the CFSE-negative MSC.

### Examination of DNA ploidy and cell fusion

DNA content per cell was determined by FACS analysis after staining the cells with propidium iodide (PI). After co-culture, cells were fixed with 70% ethanol, at 4°C for 16 hours. The cells were then stained with PI (400 ug ml-1; Sigma) just before FACS analysis. To ensure that our results were due to differentiation and not fusion of MSC with mature neurons, we analyzed the DNA content of unprimed and primed MSC before and after co-culture with mature neuronal cell lines. In all cases we did not see an increase in ploidy, suggesting that the majority of the cells were diploid and few cells were polyploid (< 2%). We also did not see any signs of cell fusion in the cells (after staining with DAPI) in any of the co-culture experiments nor in confocal analysis of the cells after transplantation in the brains of uninjured or TBI rats.

### MSC migration capability

To examine migration functionality of CXCR4 on the CD133+ABCG2+ MSC subpopulation, migration capability of these cells and the unsorted CXCR4 subpopulation was examined in response to SDF-1. For these experiments 1 × 10^5 ^elutriated but unsorted MSC, unprimed, or RA-primed CD133+ABCG2+CXCR4+ MSC were isolated from 10 blood donors. Cells were untreated, pretreated with 5 μg/ml of dexamethasone, or treated with a CXCR4 blocking antibody (Clone 44708, 10 μg/ml, R & D systems or BD) and were placed in serum-free neural basal medium without growth factors in the top chamber of a Transwell plate (Corning Life Sciences, Lowell, MA, USA) with a 5 μm pore-sized filter. A time course of migration was done in order to determine that the optimal incubation time for this assay was four hours. Lower chambers of the transwell were filled with neural basal medium containing SDF-1 (200 ng/ml), homogenates made from uninjured rat brains, TBI brain homogenate, or medium alone. Plates were incubated at 37°C, 95% humidity and 5% CO_2 _for 4 hours then cells from both chambers were collected and counted using a Multisizer 3 coulter counter (Beckman-Coulter). Results are presented as an average chemotactic index (the ratio of cells moving towards SDF-1 to the number of cells moving towards media alone) for cells isolated from 10 blood donors.

Fluid percussion injury

Animal procedures and surgeries were approved by the Institutional Animal Care and Use Committee of the University of Texas Medical Branch at Galveston and were performed under aseptic conditions in compliance with NIH Guide for Care and Use of Laboratory Animals. Non-immune-suppressed, adult, male Sprague-Dawley rats weighing 350 to 400 grams were anesthetized with isoflurane in an anesthetic chamber, intubated, and mechanically ventilated with 1.5 to 2.0% isoflurane in O_2_:room air (70:30) using a volume ventilator (EDCO Scientific, Chapel Hill, NC, USA). Rats were anesthetized and prepared for parasagittal fluid-percussion TBI as previously described [30-32]. Briefly, rats were placed in a stereotaxic frame and the scalp was sagittally incised. A 4.0 mm-diameter hole was trephined into the skull 2.0 mm to the right of the midsagittal suture and midway between the lambda and bregma. A modified Luerlok syringe hub was placed over the exposed dura, bonded in place with cyanoacrylic adhesive and covered with dental acrylic. Rats were connected to the trauma device, anesthesia was shut off and animals were subjected to moderate (2.0 ± 0.1 atm) injury. After TBI rats were disconnected from the fluid percussion device and righting reflex was assessed every 60 seconds until a normal righting reflex was observed. Rats were then again placed on 1.5% isoflurane, wound sites were treated with a topical antibiotic, infused with bupivicaine and sutured. Isoflurane was discontinued and the rats were extubated and allowed to recover in a warm, humidified incubator.

### Preparation of MSC for transplantation

CD133+ABCG2+CXCR4+ MSC were primed with RA for 24 hours before transplantation. Immediately prior to transplantation, unprimed or RA-primed CD133+ABCG2+CXCR4+ MSC were labeled with CFSE to allow for tracking of cells and estimation of MSC proliferation as described previously [33]. CFSE allows for tracking of stem cells after *in vivo *administration; 5-bromo-2-deoxyuridine (BRDU) was not used to label cells since BRDU has been shown to induce senescence [34] or be selectively toxic to MSC [35]. In a subset of experiments non-CFSE-tagged MSC were transplanted into rats used to evaluate induction of apoptosis following injury.

One day post-TBI, all animals were anesthetized with isoflurane. We used 15% Pluronic F-127 (PF-127) in DMEM as a vehicle to aid transplantation of 5 × 10^5 ^CFSE-labeled, unprimed or RA-primed CD133+ABCG2+CXCR4+ MSC, which were injected into the lateral ventricle using a 5-μl Hamilton syringe with a 26-gauge needle. Sham-injured animals underwent needle insertion and injection of PF-127 vehicle alone, or received 5 × 10^5 ^CFSE-labeled, unprimed or RA-primed CD133+ABCG2+CXCR4+ MSC, into the lateral ventricle. Animals were observed for any signs of rejection or infection until sacrificed.

### Morris water maze

The procedures for assessing spatial memory performance using the Morris Water Maze (MWM) are described in detail elsewhere [36]. Briefly, the MWM is a black tank (180 cm diameter, 28 cm depth) filled with water. A clear, Plexiglas platform was hidden beneath the surface of the water. When released, the animal swims around the pool in search of a platform. The earliest and classic measure of learning in the MWM is latency, which is the time it takes for an animal to find the platform. Beginning on post-injury day 11 (PID 11), rats were tested with four trials per day for five days. For each trial, the rat was placed in the tank facing the wall and was allowed to swim for up to 120 sec to find the hidden platform. If the rat failed to find the platform after 120 sec, it was placed on the platform by the experimenter. All rats were allowed to remain on the platform for 30 sec before being placed in a heated incubator for a four-minute rest period between trials. For the next three trials, the procedures were the same except that the rat was placed in a different quadrant of the pool. The location of the platform remained the same. After the last trial on PID 11, the platform was extended to be visible above the surface of the water. The visible platform task serves as a control for potential non-specific deficits in visual and motor function. Finally, after the last trial on the last testing day (PID 15), a single probe trial was performed. The platform was removed and the percent time in the platform quadrant was measured. Swim speed, time to find the platform and other variables were measured using a video tracking system. Normal rats will swim to the target quadrant of the pool, which is considered a good test, and TBI rats do poorly in this test and do not swim to the target quadrant. Following MWM testing animals were sacrificed, and their brains were removed and prepared for assessment of stem cell migration, differentiation and induction of apoptosis.

### Histological evaluation

Animals were anesthetized and sacrificed at 2 days, one month or three months after the administration of moderate TBI, as described in Table 2, using standard guillotine techniques. Brains were removed and fixed in 4% PF for 12 hours, embedded in paraffin or were frozen for later histological examination. Intact brains were cut in half and were then frozen in tissue freezing medium (Triangle Biomedical Sciences, Durham, NC, USA) prior to being serially sectioned on a Microm cryomicrotome (Thermo Scientific, Walsdorf, Germany). For determinations of transplanted cell survival each slide was scored by counting the total number of CFSE-positive cells in serial sections of brains from either uninjured or TBI rats that had received CFSE-labeled RA-primed or unprimed CD133+ABCG2+CXCR4+ MSC. CFSE-positive cells (green) were counted under 400× magnification. Sections from uninjured or TBI rat brains that contained CFSE-positive cells were also stained for expression of Tuj1 or NEUN. All Tuj1- and NEUN-positive cells (red) were also counted on each slide under 400× magnification. Three replicate measurements of slides were performed by the same observer for each slide. All slides were counted without knowledge of the neuronal or cell-specific marker being examined, and results were confirmed through a second reading by another person. The inter-observer agreement percentage for these cell counts was calculated and recorded.

**Table 2:** Experimental groups

Group	Treatment	Rats, number	Morris water maze-tested, number	Time of sacrifice (number)	Histology, number of rats
Sham injury	No mesenchymal stem cells (MSC)	8	8	1 month (4)	8
				3 months (4)	
Sham Injury	Primed MSC	4	Not tested	2 days (4)	4
Traumatic brain injury (TBI)	No MSC	8	8	1 month (4)	8
				3 months (4)	
TBI	No MSC	4	Not tested	2 days (4)	4
TBI	Unprimed MSC	8	8	1 month (4)	8
				3 months (4)	
TBI	Unprimed MSC	4	Not tested	2 days (4)	4
TBI	Primed MSC	8	8	1 month (4)	8
				3 months (4)	
TBI	Primed MSC	4	Not tested	2 days (4)	4

A total of 48 male Sprague-Dawley rats were randomly divided into injury or treatment groups; 12 rats were sham-injured and of these animals, 8 received no stem cell treatment and 4 received RA-primed MSC: 36 rats were subjected to moderate TBI and of these animals 12 were untreated, 12 were treated with unprimed MSC and 12 were treated with RA-primed MSC. Eight animals were randomly selected from each of these groups (sham injury, TBI, TBI with unprimed MSC, TBI treated with primed MSC) for Morris water maze (MWM) evaluation and 4 rats from this and other groups were euthanized for histological evaluations alone.

Apoptosis was measured in sections of brains from sham, TBI and TBI plus MSC-treated animals one month following injury. Apoptosis was determined by quantitation of DNA strand breaks using the terminal deoxynucleotidyl transferase dUTP nick end labeling (TUNEL) method for detecting DNA fragmentation (In Situ Cell Death Kit; Boehringer Mannheim) as described in the manufacturer's instructions. Frozen sections (5 to 7 μm) were examined for cell migration, survival and engraftment or were stained to evaluate expression of GFAP, TH, CHAT, GalC, Tuj1 and CXCR4 and human major histocompatibility molecule (MHC) class I or MHC class II (MHC II). Brain sections were also stained for the production of SDF-1 or the neuroprotective factors chondroitin sulfate proteoglycans (CSPGs) and BDNF.

### Statistical analysis

For cell phenotype analysis in Table 1, 10,000 cells were collected for each flow cytometry sample examined (RA-primed or unprimed). For all other data comparisons the paired samples *t-*test was used to compare means for 10 blood donors. Statistical analyses for these data were performed using GraphPad InSTAT software (version 2003). Mean values and SD are reported. Mean differences in the values were considered significant when *P *was < 0.05. MWM latency data were analyzed using analysis of variance for a two-factor experiment with repeated measures on time. The two factors were treatment group (4 groups) and day (5 days). Data analysis was carried out using PROC MIXED, the SAS^® ^system, release 9.1 [37], with the first order autoregressive option for covariance structure. The main effects and the interaction were tested at the 0.05 experimental-wise error rate. Multiple comparisons were conducted using Fisher's least significant difference procedure with Bonferroni adjustment for the number of comparisons.

## Results

MSC in human PB were identified by isolating small cell fractions of human MNC using counter-current centrifugal elutriation. Characterization of a 4 to 6 μm-sized elutriation fraction showed that these cells were CD105+CD90+CD29+ MSC. Subpopulations of cells were found that coexpressed CD133, ABCG2, SSEA-4 and CXCR4 (Table 1). Evaluation of the cells immediately after isolation and at 24 hours post RA priming using flow cytometry suggested that they were not of leukocyte, fibroblast, endothelial or hematopoietic stem cell lineages (Table 1). The cells were Lin-1- and CD14-negative and were not capable of phagocytosis or cytokine production prior to or after priming, or after stimulation with LPS or PHAM, which was not true for other elutriation fractions containing leukocytes isolated from the same donor (data not shown). These PB-derived MSC continued to express CXCR4 24 hours after priming, expressed low levels of human major histocompatibility complex class I (MHC I), human leukocyte antigen (HLA) A, B and C, and were negative for MHC class II (DR, DQ, DP) (Table I).

**Figure 1:**
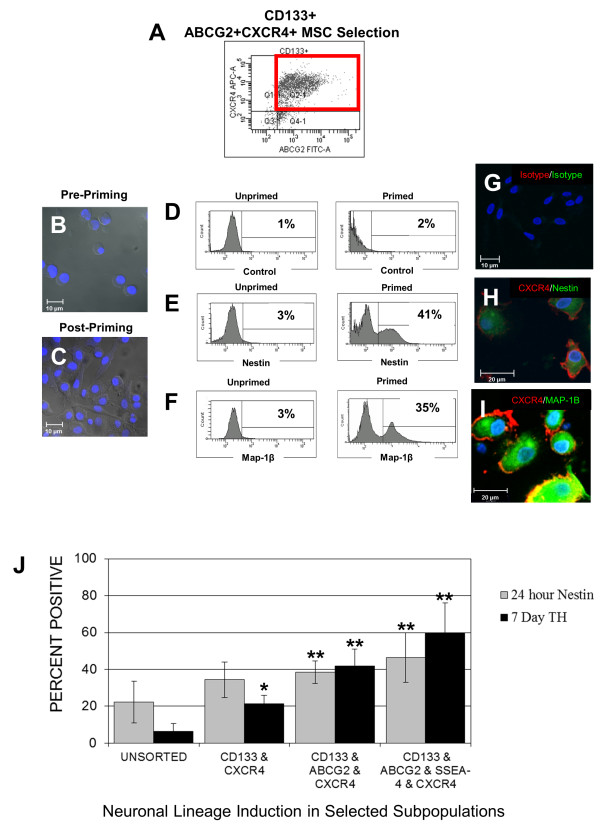
**Isolation of mesenchymal stem cells (MSC) and subpopulations**. (A) Representative dot plot for the sorting gate used in collection of CD133+ABCG2+CXCR4+ MSC. (B) Phase contrast micrograph of freshly isolated CD133+ABCG2+CXCR4+ MSC prior to, as well as (C) 24 hours post *trans*-retinoic acid (RA) priming. (D-F) A representative set of histograms showing flow cytometry evaluation of nestin and microtubule associated protein (MAP)-1β expression by unprimed or RA-primed CD133+ABCG2+CXCR4+ MSC from one donor; 10,000 cells were collected for each flow cytometry sample. (G-I) Representative confocal images of CD133+ABCG2+CXCR4+ MSC showing expression of nestin and MAP-1β at 24 hours. (G) Unprimed MSC. (H and I) RA-primed MSC; (H) sample stained for expression of CXCR4 (red) and nestin (green); (I) cells showing expression of CXCR4 (red) and MAP-1β (green). (G) H and I staining control. (J) MSC subpopulations were elutriated or sorted by fluorescence-activated cell sorting (FACS) based on co-expression of CD133 and CXCR4, CD133 and ABCG2 and CXCR4, or CD133 and ABCG2 and SSEA-4 and CXCR4. Data are for MSC subpopulations isolated from 10 blood donors. Results represent the average +/- SD. Statistically significant comparisons (*P < 0.05, **P < 0.005) for nestin and tyrosine hydroxylase (TH) production for elutriated but unsorted cells compared to each subpopulation of FACS-sorted MSC are shown.

Freshly elutriated cells did not express any neuronal lineage markers at the time of isolation (Table 1). While expression of CXCR4 remained relatively unchanged 24 hours after priming, expression of CD133, ABCG2 and SSEA-4 on MSC were significantly reduced (Table 1). An example of a sort gate used for isolation of CXCR4+CD133+ABCG2+ subpopulations is shown in Figure 1A. After elutriation, the cells were round and uniform in shape (Figure 1B). Twenty-four hours after RA priming in culture, the cells were strongly adherent to plastic and were spindle shaped with numerous cell processes (Figure 1C). The CD133+ABCG2+CXCR4+ MSC subpopulation, which was isolated from nonmobilized human PB, formed neurospheres (data not shown).

**Figure 2:**
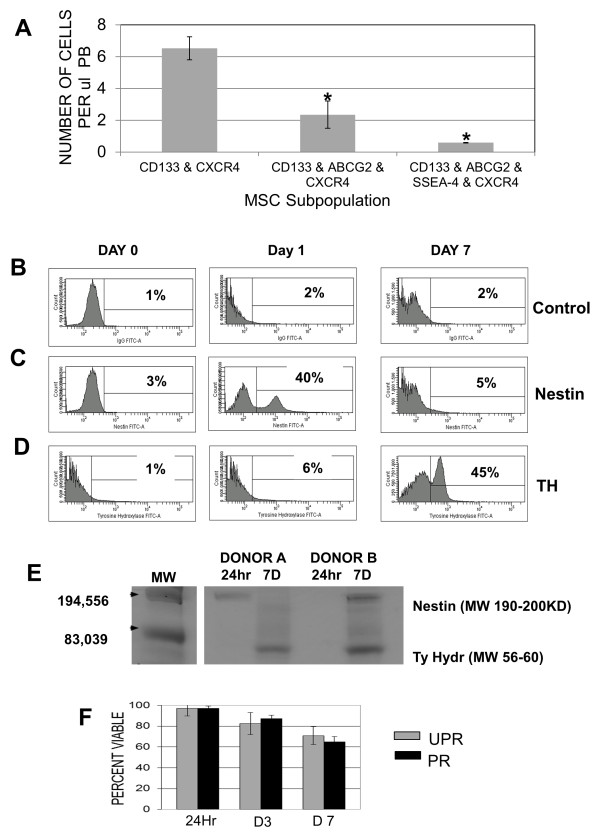
**MSC subpopulation isolation and induction of neural markers**. (A) Number of cells per μl isolated from peripheral blood (PB) for the CD133 and CXCR4, CD133 and ABCG2 and CXCR4, or CD133 and ABCG2 and SSEA-4 and CXCR4 subpopulations. Data are from 10 PB donors. Results represent the average +/- SD. Statistically significant comparisons (*P < 0.05, **P < 0.005) for the CD133 and CXCR4, or CD133 and ABCG2 and CXCR4 or CD133 and ABCG2 and SSEA-4 and CXCR4 subpopulations. (B) Representative histograms showing evaluation of CXCR4+CD133+ ABCG2+ MSC differentiation for cells isolated from one donor, pre-priming as well as 24 hours or 7 days post-RA priming; (B) isotype staining control, (C) expression of nestin, (D) expression of TH: 10,000 cells were collected for each sample. (E) Immunoprecipitation of newly produced ^35^S methionine-labeled nestin or tyrosine hydroxylase (Ty Hyd) from cells isolated from two donors (A and B); MW, molecular weight. (F) Evaluation of viability of primed or unprimed MSC isolated from 10 donors after 24 hours, 3 days (D3), or 7 (D7) days of culture.

Representative flow cytometry histograms showing staining controls (Figure 1D), expression of nestin (Figure 1E) and MAP-1β (Figure 1F) pre- and post RA priming as well as confocal images of purified control, nestin or MAP-1β-positive subpopulations (Figure 1G-I) are shown. RA-primed MSC expressed nestin (Table 1, Figure 1E and 1H) and MAP-1β (Table 1, Figure 1F and 1II) after 24 hours of culture, while unprimed cells remained undifferentiated. Both the nestin-positive and MAP-1β subpopulations continued to express CXCR4 24 hours post priming. We also examined nestin production at 24 hours and TH production at 7 days in primed subpopulations of unsorted MSC or subpopulations that were isolated based on co-expression of CD133, CD133 & ABCG2 or CD133 & ABCG2 & SSEA-4 combined with expression of CXCR4 (Figure 1J). The highest efficiency of nestin and TH production was in a CD133+ABCG2+SSEA-4+CXCR4+ subpopulation (Figure 1J). The average number of CD133+ABCG2+SSEA-4+CXCR4+ MSC isolated from 10 donors was 0.59 per ul of PB and ranged from 12,339 to 32,667 cells isolated per unit of PB (450 ml). Although the CD133+ABCG2+SSEA-4+CXCR4+ MSC subpopulation was induced to express neuronal markers more efficiently, fewer cells with this phenotype could be consistently isolated, making this population a less favorable candidate for use in our *in vivo *transplantation studies (Figure 2A). Since our goal was to evaluate the capability of selected subpopulations of CD133+ MSC to differentiate into neural lineages *in vitro *and *in vivo*, our experiments were done using a CD133+ABCG2+CXCR4+ MSC subpopulation.

**Figure 3:**
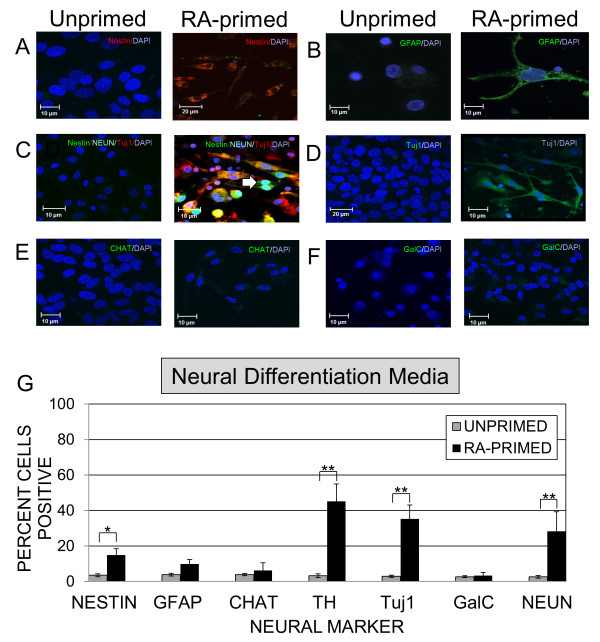
**Induction of neural markers *in vitro***. Representative confocal images of unprimed and *trans*-retinoic acid (RA)-primed fluorescence-activated cell sorting (FACS)-sorted CD133+ABCG2+CXCR4+ MSC showing expression of (A) nestin (red), (B) glial fibrillary acidic protein (GFAP) (green), (C) nestin (green), neuronal specific class III β-tubulin (Tuj1) (red) and neuron specific nuclear protein (NEUN) (teal nuclei, white arrow), (D)Tuj1 (green). For RA-primed MSC anticholine acetyl transferase (CHAT) and galactocerebroside (GalC) levels were similar to unprimed MSC. Diamidino-2-phenylindole (DAPI) nuclear stain (blue) was used in all preparations. (G) Efficiency of neuronal lineage marker expression as evaluated by flow cytometry of RA-primed MSC (solid black bars) after 7 days of culture to evaluate nestin, GFAP, CHAT, Tuj1, TH, GalC and NEUN by CD133+ABCG2+CXCR4+ MSC isolated from 10 donors, and cultured in neural differentiation media. Results represent the average +/- SD. Statistically significant comparisons indicated by brackets (*P < 0.05, **P < 0.005).

CD133+ABCG2+CXCR4+ MSC isolated from most donors expressed nestin by 24 hours and TH by 7 days (Figure 2C and 2D) although there were occasionally differences in the kinetics of expression of these proteins in cells isolated from different donors (Figure 2E). Examination of viability of unprimed and RA-primed CD133+ABCG2+CXCR4+ MSC indicated that similar levels of cell death occurred over 7 days of culture (Figure 2F). To determine the range of neural markers induced after 7 days of *in vitro *culture of CD133+ABCG2+CXCR4+ MSC in neural differentiation media, staining was performed to evaluate nestin (Figure 3A), GFAP (Figure 3B), TH and co-expression of Tuj1, nestin and NEUN (Figure 3C), Tuj1 (Figure 3D), CHAT (Figure 3E) and GalC (Figure 3F). Unprimed CD133+ABCG2+CXCR4+ MSC remained unchanged in morphology after 7 days of culture and did not express mature neuronal markers (Figure 3A-F and 3G). Primed CD133+ABCG2+ CXCR4+ MSC became extremely elongated and produced multiple cell processes (Figure 3A-D). After 7 days of culture, less than 3.5% of unprimed MSC spontaneously expressed mature neural markers, but RA-primed CD133+ABCG2+CXCR4+ MSC consistently showed increased TH (45.2 +/- 9.7%), Tuj1 (35.23 +/-7.9%) and NEUN (28.2 +/-.11.1%) (Figure 3G). Expression of GFAP was low in these experiments (4.88 +/- 2.4%) and was slightly higher than for unprimed MSC (3.49+/- 0.7%). CHAT and GalC expression were the same as background levels of the unprimed MSC subpopulation (< 3%) (Figure 3E-G).

### Co-culture experiments

**Figure 4:**
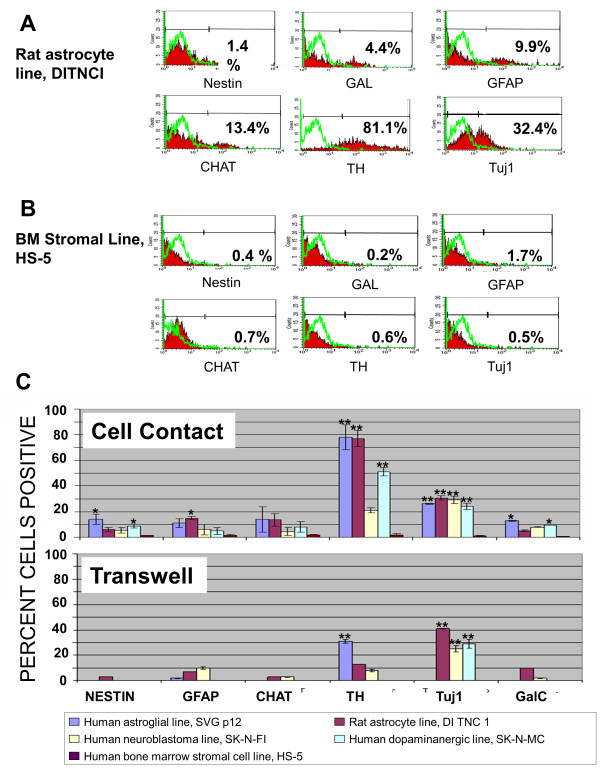
**Microenvironmental cues and neuronal marker expression**. (A) Flow cytometry histograms showing neuronal marker expression by *trans*-retinoic acid (RA)-primed CD133+ABCG2+CXCR4+ MSC after co-culture allowing direct cell to cell contact with the rat astrocyte cell line (DITNC1). (B) Co-culture of RA-primed CD133+ABCG2+CXCR4+ MSC allowing cell to cell contact with human bone marrow cell line (HS-5). Isotype staining control for each is shown as a green overlay histogram. Control levels for all samples were ≤ 2% cells positive. (C) RA-primed CD133+ABCG2+CXCR4+ MSC were cultured allowing cell to cell contact (top graph) or no contact (bottom graph) with human astroglial (SVGp12), human neuroblastoma (SK-N-F1), rat astrocyte (DITNC1), human dopaminergic (SK-N-MC) cell line or, as a control, HS-5. Efficiency of neuronal marker expression after 7 days of culture for nestin, glial fibrillary acidic protein (GFA), anticholine acetyl transferase (CHAT), tyrosine hydroxylase (TH), type III beta tubulin (Tuj1) and galactocerebroside (GalC) production is shown. Results represent the average +/- SD for 10 donors. Statistically significant comparisons (*P < 0.05, **P < 0.005) are indicated for each marker compared to control cells, which were RA-primed but not cultured with any of the cell lines listed. Unprimed cells expressed < 2% positive cells when cultured with mature neural cells for all of the mature neuronal markers evaluated.

For co-culture experiments allowing direct cell to cell contact, neuronal cell lines were labeled with CFSE prior to addition to the culture of unlabeled, unprimed or RA-primed CD133+ABCG2+CXCR4+ MSC. Analysis was done by gating on the CFSE-negative CD133+ABCG2+CXCR4+ MSC population and sorting the MSC prior to staining for expression of mature neuronal markers. Flow cytometry analysis for a representative co-culture experiment allowing cell contact between RA-primed MSC and the CFSE-labeled rat astrocye cell line, DITNCI, is shown in Figure 4A. In this experiment TH was induced in 81.1% of the primed MSC after 7 days (Figure 4A) in the absence of cell fusion with pre-existing mature neuronal cells. Only 0.6% of primed CD133+ABCG2+CXCR4+ MSC were induced to express TH after co-culture with the bone marrow stromal line (HS-5) (Figure 4B). Isotype control staining was 2% +/- 0.92 or less for both cultures and is shown as the green plot overlay (Figure 4A and 4B). Direct contact co-culture with human astroglial (SVGp12) cells induced high levels of expression of TH as did co-culture with rat astrocyte (DITNC 1) cells and dopaminanergic (SK-N-MC) cells (Figure 4C), but induced lower levels of other neural markers (Figure 4C, top graph).

When CD133+ABCG2+CXCR4+ MSC were cultured in transwells that did not allow cell to cell contact the highest levels of neural marker induction also occurred when MSC were cultured with human astroglial (SVGp12) cells, which induced high levels of expression of TH as did co-culture with rat astrocyte (DITNC 1) or the neuroblastoma line (SK-N-FI) (Figure 4C, bottom graph). Differentiation efficiency of cells in transwell cultures was much lower than that of cells with direct contact. Neither unprimed CD133+ABCG2+CXCR4+ MSC nor RA-primed MSC cultured with the bone marrow stromal line HS-5 induced production of the neuronal markers when directly cultured with MSC or in transwell culture allowing no direct cell to cell contact (Figure 4C). To ensure that our results were due to differentiation, and not to fusion of CD133+ABCG2+CXCR4+ MSC with mature neurons we analyzed the DNA content of unprimed and RA-primed MSC before and after co-culture with mature neuronal cell lines. In all cases we did not see an increase in ploidy suggesting that the majority of the cells were diploid and few cells were polyploid (< 2%). We also did not see any signs of cell fusion (after staining with DAPI) in any of the co-culture experiments, nor in confocal analysis of the cells after transplantation into the brains of uninjured or TBI rats.

### Chemotaxis assay

**Figure 5:**
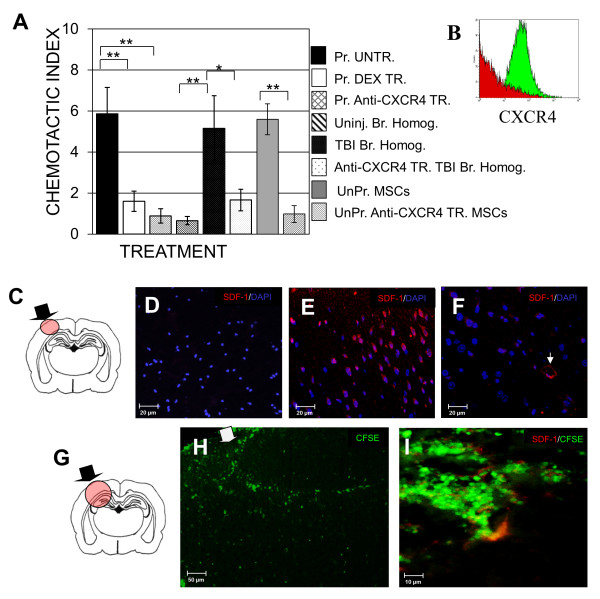
**Migration capacity of mesenchymal stem cells (MSC)**. (A) Evaluation of the migration ability of CD133+ABCG2+CXCR4+ MSC. Results are presented as a chemotactic index (the ratio of cells moving towards stromal cell-derived factor-1 (SDF-1) or tissue homogenates compared to the number of cells moving towards media alone). Untreated (UNTR.), dexamethasone-treated (DEX TR.) and CXCR4 antibody-treated (ANTI-CXCR4 TR.) *Trans*-retinoic acid (RA)-primed MSC were evaluated for their ability to migrate. Migration of this cell population was also evaluated in response to uninjured (Uninj.) or injured (TBI) brain homogenates (Br. Homog.) alone or brain homogenate after treatment of MSC with anti-CXCR4 blocking antibody. Results for the migration of elutriated but unsorted MSC with and without treatment with anti-CXCR4 blocking antibody are also shown. Results represent the average +/- SD in the chemotactic index for 10 blood donors. Statistically significant comparisons are indicated in brackets (*P < 0.05, **P < 0.005). (B) Evaluation of CXCR4 expression before (green histogram) and after dexamethasone treatment of MSC (red histogram) using quantitative flow cytometry. (C) Schematic showing both the injury site (black arrow) and general regions evaluated in both uninjured and TBI rats shown in D-I (red circle). (D, E, F) Production of SDF-1 in (D) uninjured rat, (E) TBI rat at 2 days and (F) at 1 month post injury. Arrow points to positive staining for SDF-1 in a micro-vessel in the brain 2 days after transplantation of MSC. (G) Schematic showing both the injury site (black arrow) and general regions evaluated in both H and I. (H) Migration of CFSE+ CD133+ABCG2+CXCR4+ MSC. Carboxyfluorescein succinimidyl ester (CFSE)-labeled cells, green, (white arrow injury site) and (I) production of SDF-1 (red) in TBI rats as well as presence of CFSE-labeled MSC after 1 month; (D-I) 4',6-diamidino-2-phenylindole (DAPI) blue nuclei.

Success of a cell therapy depends on sufficient recruitment of applied cells to the injury site or target tissue. Unprimed and RA-primed CD133+ABCG2+CXCR4+ MSC could be shown to migrate in response to SDF-1 in transwell migration assays (Figure 5A). Dexamethasone treatment of unprimed and RA-primed CD133+ABCG2+CXCR4+ MSC reduced CXCR4 expression and subsequent migration of these cells *in vitro *(Figure 5A and 5B, red, dexamethasone-treated; green, untreated). Migration in these assays was also reduced after anti-CXCR4 antibody treatment. RA-primed CD133+ABCG2+CXCR4+ MSC migrated in response to homogenized brains of TBI rats but not in response to homogenized brains from uninjured rats (Figure 5A) and migration in response to TBI brain homogenate could be blocked by addition of anti-CXCR-4 antibody (*P *= 6.5 × 10^-6^) supporting a CXCR4-mediated mechanism of cell migration for this cell subpopulation. Elutriated but unsorted CXCR4+ MSC were also shown to migrate in response to SDF-1 (Figure 5A) and the migration of this cell population was also blocked using anti-CXCR4 blocking antibody (Figure 5A and 5B).

The injury site and areas corresponding to the location of the tissue sections are shown in Figure 5C. Following *in vivo *transplantation of CD133+ABCG2+CXCR4+ MSC into the lateral ventricle of uninjured and TBI animals, brain sections were stained for the presence of SDF-1 (Figure 5D-F and 5I). Sections of brains from uninjured animals did not show staining for SDF-1 and few CFSE+ cells were found to have migrated to the regions corresponding to the injury site in these animals (Figure 5D). Sections of brains from TBI rats show the presence of high levels of SDF-1 (Figure 5E, F and 5I) at the injury site and numerous CFSE+ cells were seen to have migrated into these high-SDF-1 + areas (Figure 5H and 5I).

### MWM latency results

**Figure 6:**
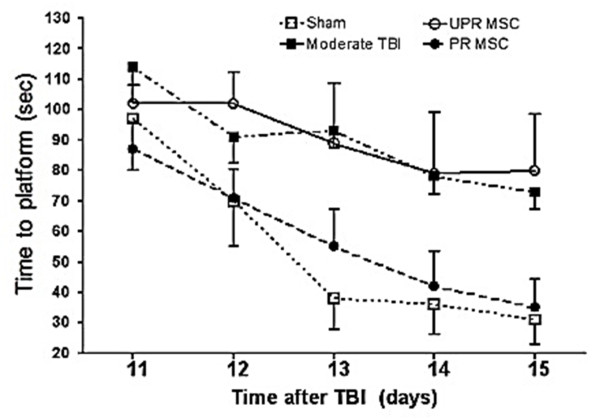
**Morris water maze (MWM) test**. (A) Time to find a hidden platform in the MWM (latency) in rats subjected to no injury (sham) or moderate traumatic brain injury (moderate TBI) alone, or TBI followed by transplantation with unprimed (UPR) or *trans*-retinoic acid (RA)-primed (PR) CD133+ABCG2+CXCR4+ MSC. Latency of the TBI-alone and TBI + unprimed MSC groups were not statistically significantly different. However, primed MSC after TBI improved the latency to the level of uninjured group.

Interaction of treatment group by day was not statistically significant. That is, treatment group differences did not statistically significantly fluctuate from day to day, or they remained the same from day to day (Figure 6). Overall (across all time points) latency of the TBI-alone group and the TBI+ unprimed MSC group were not statistically significantly different. That is, unprimed MSC did not significantly improve the overall latency after TBI and remained at the level of the TBI-alone group. Overall latency of the TBI primed MSC group and uninjured group were not significantly different. That is, primed MSC after TBI improved the latency to the level of the uninjured group. For overall latency, there were differences between the TBI-alone and the uninjured group, and between the TBI+ unprimed MSC and TBI+ primed MSC groups, although these were not statistically significant. Overall (across the four treatment groups) latency at day 12 was statistically significantly improved from day 11. Similarly, overall latency at day 13 was significantly improved from day 12. Overall latency from day 13 to day 14, and also day 14 to day 15 continued to improve, although this was not statistically significant.

### Histological examination at 2 days or 1 month post transplantation

**Figure 7:**
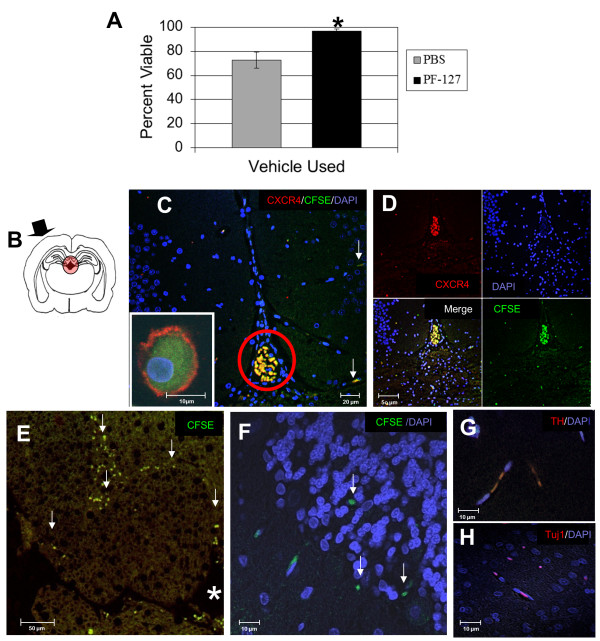
**Examination of cells pre-transplantation and 2 days or 1 month post transplantation**. (A) Viability of *trans*-retinoic acid (RA)-primed CD133+ABCG2+CXCR4+ mesenchymal stem cells (MSC) held in either PBS or pluronic F-127 (PF-127) carrier for 2 hours prior to implantation. Results represent the average +/- SD for viability of cells from 10 blood donors. Statistically significant comparisons (*P < 0.05) for PBS compared to PF-127 as vehicle are indicated. (B) Schematic diagram showing site of implantation of MSC into the lateral ventricle (red circle) of non-immunosuppressed male Sprague-Dawley rats. Arrow indicates the site of the fluid percussion traumatic brain injury (TBI). (C-H) Confocal micrographs of 7-μm sections of brains. (C) Sections from uninjured rats sacrificed at 1 month showed that carboxyfluorescein succinimidyl ester (CFSE)-labeled MSC (green) expressed CXCR4 (red). White arrows point to CFSE-labeled MSC. (D) Sections of brains of TBI rats sacrificed 2 days after transplantation. CXCR-4+ CD133+ABCG2+CXCR4+ MSC are noted with white arrows. (E) Brain section 1 month after transplantation (ventricle is indicated by the white *, white arrows identify migrated CFSE+ (green) MSC. (F) Higher magnification image of MSC in the hippocampus of the TBI rat. (G) Some cells were elongated and expressed tyrosine hydroxylase (TH) (red) or (H) type III beta tubulin (Tuj1) (red). (C-H) diamidino-2-phenylindole (DAPI) nuclear stain (blue).

CD133+ABCG2+CXCR4+ MSC were held at 4°C for 2 hours prior to transplantation. Viability of MSC at 4°C in 15% PF-127 in DMEM was consistently better than in PBS (Figure 7A), and because of this 15% PF-127 in DMEM was used as a vehicle for transplantation. CFSE-labeled MSC were placed in the lateral ventricle (Figure 7B, black arrow injury site). In uninjured rats one month after transplantation of CD133+ABCG2+ CXCR4+ MSC only a small fraction of RA-primed MSC migrated out of the ventricle into regions such as the cortex and hippocampus (Figure 7C). Most MSC in the ventricle were amoeboid in shape and expressed CXCR4 (Figure 7C and 7D). As early as 2 days post transplantation in rats subjected to TBI, more cells were seen to have migrated out of the ventricle than had occurred in uninjured rats by 1 month (Figure 7C compared to 7D). In TBI rats sacrificed after one month few CD133+ABCG2+ CXCR4+ MSC remained in the lateral ventricle, and the majority of these cells moved to regions closer to the injury site (Figure 7E and 7F). Many MSC were found in the hippocampus (Figure 7F) and cortex (Figure 7G and 7H) and some of these cells expressed TH (Figure 7G) as well as Tuj1 (Figure 7H).

**Figure 8:**
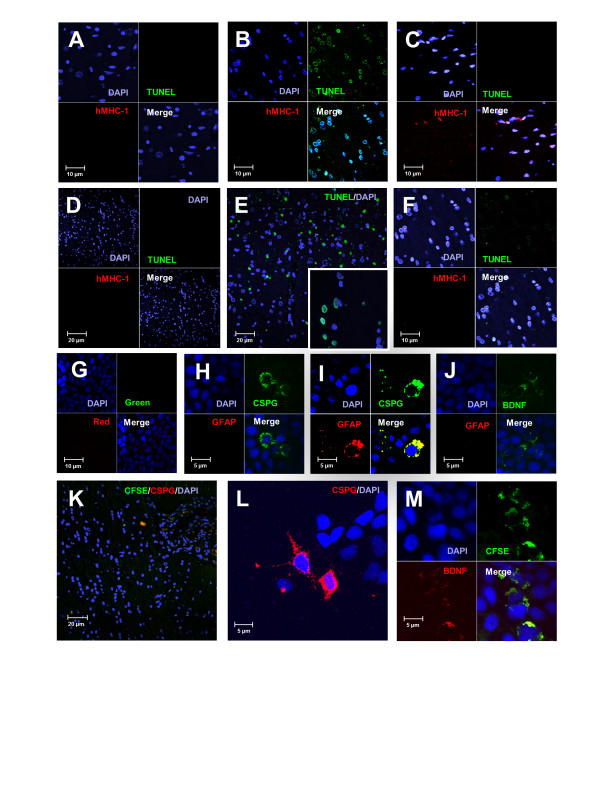
**Examination of induction of apoptosis**. Brains were harvested at 2 days (A-C) or 1 month (D-F) and subjected to terminal deoxynucleotidyl transferase dUTP nick end labeling (TUNEL) analysis. Representative images of sections taken from (A) uninjured animals, (B) traumatic brain injury (TBI) animals and (C) TBI animals that received *trans*-retinoic acid (RA)-primed mesenchymal stem cells (MSC). Evaluation of apoptosis induction one month following TBI is shown in (D) uninjured (E) TBI animals and (F) TBI animals that received RA-primed MSC. Insert in E shows higher power image of TUNEL+ nuclei. Production of chondroitin sulfate proteoglycan (CSPG), glial fibrillary acidic protein (GFAP) or brain derived neurotropic factor (BDNF) in (G) unprimed MSC and (H-J) RA-primed MSC cultured for 7 days *in vitro*. (H) Staining for expression of CSPG (green) in cells not expressing GFAP (red) in RA-primed MSC. (I) Co-expression of CSPG (green) and GFAP (red) in some RA-primed MSC. (J) Expression of BDNF (green) and GFAP (red) in RA-primed MSC. (K-M) Evaluation of CSPG and BDNF in RA-primed MSC *in vivo *following TBI. (K) Evaluation of MSC products in sections of brains harvested at 2 days post transplantation of carboxyfluorescein succinimidyl ester (CFSE)-tagged MSC in regions near the injury site, CFSE-positive MSC (green) and CSPG (red). (J) Cells positive for CSPG and diamidino-2-phenylindole (DAPI) and (M) cells positive for CFSE (green) and BDNF (red) also seen in regions near the injury site. (A-M) DAPI nuclear stain (blue).

In a subset of experiments non-CFSE-tagged MSC were transplanted into rats used to evaluate induction of apoptosis using the TUNEL assay. Examination of levels of apoptosis at the injury site in these animals showed that no apoptosis was seen in uninjured rats at 2 days (Figure 8A), or at 1 month post transplant (Figure 8D). In TBI animals, apoptotic nuclei could be seen in sections near the injury site at 2 days (Figure 8B) but few apoptotic cells were seen in MSC-treated TBI animals and staining for human MHC I showed the presence of cells in this area (Figure 8C). Similar results were seen at 1 month with many apoptotic nuclei present in regions near the injury site with less apoptosis seen in the in the TBI and MSC-treated group than in TBI animals that did not receive transplanted MSC (Figure 8E and 8F).

Evaluation of MSC products in sections of brains harvested at 2 days post transplantation of CFSE-tagged MSC in regions near the injury site showed production of CSPG (Figure 8H), GFAP (Figure 8I) and BDNF (Figure 8J), which was seen for RA-primed MSC cultured for 7 days *in vitro *but not in unprimed MSC (Figure 8G); evaluation also showed production of CSPG (Figure 8K and 8M) as well as BDNF(Figure 8M) in regions near the injury site.

### MSC survival, location and fate three months post transplantation

**Figure 9:**
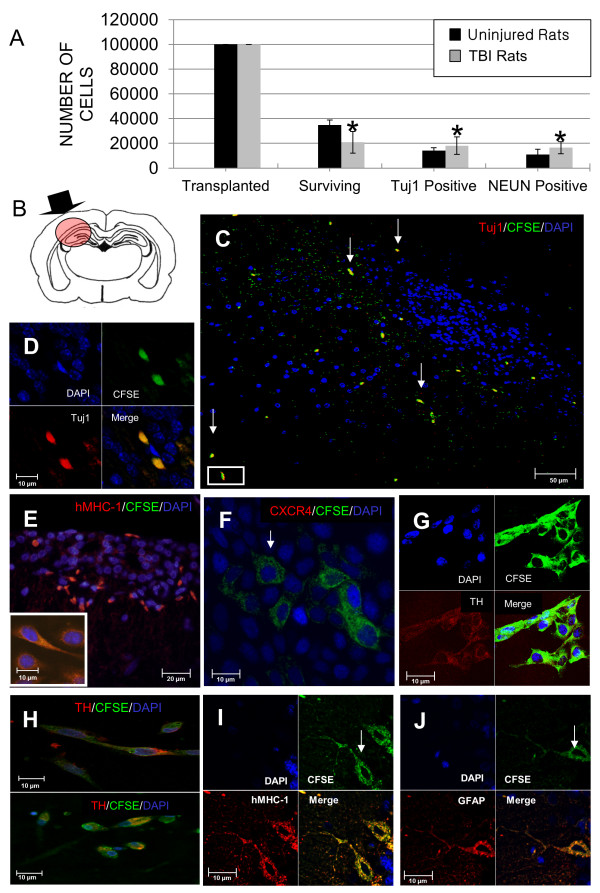
**Evaluation of mesenchymal stem cells (MSC) 3 months post transplantation**. (A) Results represent the average +/- SD for cell counts from brains of uninjured (*n *= 8) and traumatic brain injury (TBI) (*n *= 8) rats. Statistically significant comparisons (*P < 0.05) for expression of type III beta tubulin (Tuj1) or neuron specific nuclear protein (NEUN) in brain sections from uninjured compared to TBI rats. (B) Diagram of locations viewed in images B-I (red shaded circle) and location of site of fluid percussion injury (black arrow). (C) Brain section from TBI rats sacrificed 3 months after transplantation of carboxyfluorescein succinimidyl ester (CFSE)+ CD133+ABCG2+CXCR4+ MSC showing cells in the cortex and hippocampus near the injury site. White arrows indicate CFSE-positive MSC (green). (D) Image of area outlined in box of figure B, CFSE (green) and Tuj1 (red). (E-J) Examination of transplanted MSC showed that most cells had elongated, CFSE (green), DAPI (blue). (E) These cells were positive for expression of human major histocompatibility complex class I (MHC 1) (red). (F) CFSE+ cells in the hippocampus at 3 months had reduced levels of CXCR4 staining. (F and G) In some regions of the hippocampus, networks of MSC showed interconnections between cells and many cells in these clusters were (G and H) tyrosine hydroxylase (TH)-positive. (I) Human MHC I expression by cells in J and (J) a small portion of MSC were positive for expression of the astrocyte marker GFAP, and (H) had a more complex morphology with multiple branched cell processes. (D-J) Diamidino-2-phenylindole (DAPI) nuclear stain (blue).

Examination of cell survival after transplantation indicated that 34% of cells transplanted into uninjured rats survived until 3 months compared to 21% survival in TBI rats (Figure 9A). Of the cells surviving transplantation, significantly more cells in the TBI rats were positive for expression of Tuj1 (87%) and NEUN (91%) compared to expression of Tuj1 (41%) or NEUN (31%) in uninjured rats (Figure 9A). The inter-observer agreement for the cell counts in uninjured rats was 100% for counts of CFSE+ cells, 99% for Tuj1 counts and 100% for NEUN counts, and in TBI rats was 100% for counts of CFSE+ cells, 100% for Tuj1 counts and 100% for NEUN. A diagram of the injury site (Figure 9B) shows the regions examined in Figure 9C-J. Morphology of the CD133+ABCG2+CXCR4+ MSC engrafted in the brain after 3 months included amoeboid, elongated or rod-shaped cells (Figure 9C-H), as well as cells with more complex morphology (Figure 9I and 9J). Many cells in regions shown in Figure 8C were positive for expression of Tuj1 (Figure 9D). Many elongated, TH-expressing cells were found in the hippocampus (Figure 9F-H). MHC I staining confirmed that the CFSE+ cells were human in origin (Figure 9E and 9I). In some regions of the hippocampus networks of MSC showed interconnections between cells (Figure 9F-H) and many of the cells in these clusters of MSC were shown to be TH-positive (Figure 9G and 9H). A small portion of MSC was positive for GFAP, an astrocyte marker, and had a more complex morphology with multiple branched cell processes (Figure 9J).

## Discussion

Previous studies have shown the benefit of using MSC for the treatment of ischemia or TBI in animal models [18,4,6] but investigations to quantify MSC neural differentiation efficiency have yet to be accomplished. In order to realize the clinical benefits of MSC and other stem cell types, consistent methods for MSC subpopulation selection and higher efficiencies of differentiation towards neural lineages need to be realized. Stem cells generally require lineage priming in order to express genes associated with commitment to specific differentiation pathways. RA treatment has been used for priming embryonic [38] and adult stem cells for neuronal marker induction, development of neuronal lineages [25-28] and to promote survival, proliferation and neurotropic responsiveness [27,28]. It is important to consider that although priming has been shown to initiate neurogenesis or neuronal marker induction, it does not guarantee production of a specific neuronal lineage and this remains a major hurdle to development of any neural stem cell therapy.

In this study we were interested in assessing the neural lineage potential of subpopulations of MSC that could be capable of migrating to injury sites based on the CXCR4/SDF-1 pathway. We focused our attention on the isolation of HPBD MSC populations that co-expressed immature stem cell markers CD133, as well as ABCG2 or SSEA-4 combined with expression of CXCR4.

MSC were isolated using centrifugal elutriation. We then sorted the MSC based on expression of CD133, or CD133 and ABCG2, or CD133 and ABCG2 and SSEA-4 combined with expression of CXCR4. We examined the capability of each MSC subpopulation to express neuronal markers following RA priming. Addition of expression of each subsequent marker used at the time of isolation resulted in a stepwise increase in efficiency of neuronal marker induction. However, the yield of cells for each subpopulation as cell surface markers were added was significantly reduced. One subpopulation of CXCR4+CD133+ABCG2+SSEA-4+ MSC had the highest efficiency of neural marker induction as measured by expression of nestin at 24 hours and TH by 7 days of culture but we were not able to consistently isolate enough of these cells to allow for extensive *in vitro *or *in *vivo study. It is because of this that we continued our work using CD133+ABCG2+ CXCR4+ MSC. Although isolation of adequate cell numbers for use as a therapy is important, the efficiency with which MSC are able to differentiate into specific lineages is perhaps the critical factor when considering a cell population for use as a cell-based therapy. In many disease processes, it is conceivable that a small number of cells that efficiently differentiate into a specific lineage and migrate to sites of injury would be more effective than larger numbers of less efficient cells. The evaluation of subpopulations such as the ones described here form the basis for exploring co-expression of other markers allowing for enrichment of cell types that consistently differentiate into specific neuronal lineages, which might prove to be of greater therapeutic value.

This subpopulation of CD133+ABCG2+CXCR4+ MSC required RA priming to initiate induction of early neuronal markers nestin and MAP-1β, and later the expression of more mature neuronal markers such as TH and GFAP. Neural lineage markers that were used to evaluate the neural potential of subpopulations of CD133+ABCG2+CXCR4+ MSC included nestin, MAP1-β, TH, Tuj1, NEUN, GFAP, CHAT and GalC.

In our *in vitro *experiments CD133+ABCG2+CXCR4+ MSC consistently produced low percentages of cells (< 10%) expressing neuronal markers such as CHAT, GalC, GFAP but higher percentages of cells expressing TH (45.2 +/- 9.7%), Tuj1 (35.23 +/- 7.9%) and NEUN (28.2 +/- 11.1%). *In vitro *assays also showed that the CD133+ABCG2+CXCR4+ subpopulation of cells isolated from a variety of donors could be consistently directed at high efficiency towards production of TH. Similar results were observed when CD133+ABCG2+CXCR4+ MSC were co-cultured with mature neuronal lines in either mixed cultures that allowed for direct cell contact, or a transwell system, which only allowed for transfer of soluble factors (Figure 3). Co-culture of CD133+ABCG2+CXCR4+ MSC with human astroglial (SVGp12) cells induced high levels of expression of TH as did co-culture with rat astrocyte (DITNC 1) cells and dopaminanergic (SK-N-MC) cells (Figure 3). In all cases of *in vitro *culture morphological and immunophenotypic changes suggestive of neuronal differentiation persisted over 7 days.

One striking feature of CD133+ABCG2+CXCR4+ MSC *in vitro *differentiation was the speed of the response, since within 12 hours (for most donor cells) and consistently by 24 hours after priming, MSC assumed features typical of neuronal morphology in both *in vivo *and *in vitro *culture. It is our feeling that the faster the cells can be isolated, primed and transplanted within 24 hours after the injury the better, since early treatment could limit damage to the brain, subsequent loss of neurons, and allow MSC to participate in the healing process.

Our data also support the critical role played by the CXCR4/SDF-1 pathway in migration of CD133+ABCG2+CXCR4+ MSC towards regions of brain injury [20-22]. MSC migrated *in vitro *in response to SDF-1 and also migrated in response to supernatants produced from the homogenized brains of TBI rats but not uninjured rats (Figure 4A). Loss of ability to migrate after dexamethasone treatment due to reduced expression of CXCR4 has been previously described [39]. CD133+ABCG2+CXCR4+ MSC treated with dexamethasone showed reduced levels of CXCR4 and exhibited a significant reduction in the chemo-attractant and transmigration effect of SDF-1 *in vitro*. These data suggest that dexamethasone might decrease the migratory capability of MSC *in vivo *if given during the acute phase of a TBI. Transplantation of primed MSC into the brains of uninjured rats did not result in high levels of migration and most cells remained in the lateral ventricle one month after transplantation. Primed CXCR4+MSC migrated to the injury site in TBI rats and into regions also positive for SDF-1.

In order to evaluate the influence of the *in vivo *microenvironment or the atypical and potentially inhospitable environment created by TBI on MSC graft survival, migration and differentiation, we transplanted MSC into the brains of either uninjured or TBI rats. We delivered human CD133+ABCG2+CXCR4+ MSC directly into the lateral ventricle of rat brains 24 hours after fluid percussion injury. This was done to take advantage of a brain access point created by the current clinical practice of placing an intra-ventricular catheter in the lateral ventricle for measuring intracranial pressure and therapeutically removing cerebrospinal fluid in patients with moderate to severe TBI. It is important to note that delivery of primed MSC in a clinical setting could also be done through an intraventricular route, thereby reducing the need to directly place the stem cells at an injury site. We also utilized this transplantation site so that MSC could use the normal migratory pathway used by neuroblasts from the subventricular zone (SVZ) traveling along the lateral ventricles via the flow of CNS fluid [40], and be in a better position to respond to the brain's own injury-induced chemokine-assisted migration cues. This was also done because evidence suggests that the cells of the SVZ provide an environment that normally promotes neurogenesis [41].

Several studies have documented an acute injury period during which release of inflammatory factors is extremely robust, creating a hostile environment for cell engraftment and differentiation followed by a fall in the release of injury response factors over time [42-46]. This might be an explanation for those MSC that retained CXCR4 expression which, although they had migrated to the injury site, remained quiescent with no sign of morphologic indication of differentiation. Injured brains of experimental animals in other studies receiving fluid percussion TBI exhibited both primary as well as secondary (delayed) neuronal death for a prolonged period after injury, and it has been shown that neural degeneration occurs as early as 10 minutes after injury [45,32].

Analysis of rat brains 3 months after transplantation of CD133+ABCG2+CXCR4+ MSC showed that many cells had lost expression of CXCR4, differentiated and produced Tuj1, TH as well as GFAP and NEUN. We did find it interesting to note that although more CFSE+ cells were found in uninjured animals at 3 months than in TBI rats, far more cells in TBI rats were positive for expression of Tuj1 (87% compared to 41%) and NEUN (91 compared to 77%). Expression of Tuj1 is indicative of immature neurons but not necessarily integration of MSC into the brain, but the expression of NEUN indicates the presence of mature neurons that integrated into the brain. Differences in the expression of markers of neuronal maturation of the MSC *in vivo *may be due to the site at which the cells engrafted, but it is also important to consider that the influence of factors produced in response to the injury could potentially override cues to differentiate in a site-specific manner.

Functional evaluation of the use of MSC treatment in TBI animals to examine spatial memory performance using the MWM showed that improvement was seen in MSC-treated compared to untreated TBI rats. The effect of TBI on MWM latency was practically significant compared with the sham group. Primed MSC after TBI considerably improved MWM latency compared with the unprimed MSC group. However, those effects were not statistically significant due to small numbers of animals in each group and large variability in the data. Assuming the variability in the data and the mean differences remain the same or remain similar, we would have needed 10 to 11 animals per group to detect a difference at the 0.05 level of significance and a power of 80%. Isolation of adequate numbers of human stem cells for transplantation limited the numbers of animals that could be used in these experiments.

Despite the lack of maturation of the majority of large numbers of MSC after transplantation, we did see improvement in the cognitive ability of TBI rats over the testing period, suggesting that neural primed MSC may function as supportive cells in concert with endogenous stem cells from the hippocampus and SVZ of the lateral ventricle in the reparative process.

MSC have been shown to have limited survival after engraftment in the CNS of TBI rats [42,43]. In an attempt to reduce graft cell loss following transplantation we chose to utilize the hydrogel Pluronic (PF)-127 as a cell transport vehicle. PF-127 forms a soft gel at room temperature, making transplantation easier [47], and several reports have documented the ability of PF-127 to stabilize membranes [48-50]. These studies demonstrated the potential for hydrogels as a clinically feasible delivery system. Poloxamer hydrogels, such as pluronic-188, have also been shown to reduce tissue damage and macrophage infiltration in rats following TBI, thus, reducing inflammation [50]. It is important to note that although we did not use any immunosuppressive therapy, nor irradiate human MSC prior to transplantation, we did not see any signs of inflammation or leukocyte infiltration into the brain tissue in sham, TBI or MSC-treated rats.

The therapeutic effects of MSC following transplantation can also be due to inhibition of pathogenic immune responses and release of neuroprotective factors [51-59] or immunomodulatory molecules [52-55,57,59,60]. We feel that the improvement in cognitive function, measured by the MWM demonstrated by TBI animals treated with RA-primed MSC in this study at early time points, such as at 2 days, was not due to MSC replacement of damaged cells at the injury site. Examination of the results suggest that the early influence of the transplanted cells was more likely due to release of factors such as CSPG, BDNF or other as yet undefined factors, which may have helped to support damaged cells at the injury site and reduce apoptosis. We did see some differentiation of the transplanted cells as indicated by Tuj1 or TH expression at 1 month, as well as GFAP production, which is indicative of astrocyte formation at 3 months but the majority of transplanted cells did not express mature neuronal markers at the 2-day time point. Future studies will concentrate on evaluation of MSC-derived immune or neuroprotective factors as well as evaluation of factor production of MSC subsets.

## Conclusions

In summary, the practical design of any stem cell-based clinical therapy should include: (1) identification of cell surface markers that allow for consistent isolation of cells in sufficient numbers and purity for use in a therapy; (2) differentiation at high efficiency to neural lineages; (3) consideration of the ability of the cells to migrate to damaged areas without the need for direct transplantation at the site where the injury has occurred, and (4) consideration of the ability of cells to produce neuro-protective factors. In this study we provide important information about the development of potential therapeutic strategies to enhance the clinical applicability of MSC transplantation. We demonstrate here that a population of human CD133+ABCG2+CXCR4+ MSC could be consistently isolated from nonmobilized PB and induced to produce factors that support cell survival at early time points following transplantation, and to differentiate along neural lineages after RA priming. Our findings have implications for the development of clinical strategies to examine the effectiveness and applicability of the use of PB-derived MSC for the treatment of TBI or other neurodegenerative diseases. As methods for identification and isolation of adult MSC improve, the ability to uncover subpopulations whose lineage determinants combined with priming strategies target the production of a specific neuronal cell type, cell population or neuro-protective factor production, may enhance our ability to develop stem cell-based therapeutics in the future.

## Abbreviations

ABCG2: ATP-binding cassette sub-family G member 2; ANOVA: analysis of variance; BDNF: brain derived neurotropic factor; β-ME: β-mercaptoethanol; CFSE: carboxyfluorescein succinimidyl ester; CHAT: choline acetyltransferase; CNS: central nervous system; CSPG: chondroitin sulfate proteoglycan; CXCR4: C-X-C chemokine receptor type 4; DAPI: diamidino-2-phenylindole; DMEM: Dulbecco's modified Eagles medium; EGF: epidermal growth factor; FACS: fluorescence activated cell sorting; FGF: fibroblast growth factor; FITC: fluorescein isothiocyanate; GalC: galactocerebroside; GFAP: glial fibrillary acidic protein; HPBD: human peripheral blood-derived; HLA: human leukocyte antigen; IgG: immunoglobulin; IL: interleukin; INF-γ: interferon-gamma; Lin-1: lineage-1; LPS: lipopolysaccharide; MAP-1β: microtubule associated protein 1β; MHC: major histocompatibility complex; MNC: mononuclear cells; MSC: mesenchymal stem cells; MWM: Morris water maze; NDM: neural differentiation media; NEUN: neuron specific nuclear protein; PB: peripheral blood; PBS: phosphate buffered saline; PE: phycoerytherin; PF-127: pluronic F-127; PF: paraformaldehyde; PHAM: phytohemagglutinin-M; PID: post-injury day; PNS: peripheral nervous system; RA: *trans*-retinoic acid; SDF-1: stromal derived factor-1; SSEA-4: stage-specific embryonic antigen-4; SVZ: subventricular zone; TBI: traumatic brain injury; TBS: tris buffered saline; TH: tyrosine hydroxylase; TNF: tumor necrosis factor; Tuj1: type III beta tubulin; TUNEL: terminal deoxynucleotidyl transferase dUTP nick end labeling.

## Competing interests

The authors declare that they have no competing interests.

## Authors' contributions

JEN and JC were responsible for conception and design of the experiments, data collection, assembly, interpretation, manuscript writing and final approval of the manuscript. JAN, MP, AC, EL and SV were responsible for data collection, data analysis and generation of the methods sections. DW and DP were responsible for design and completion of animal studies and MWM testing as well as final approval of the manuscript. All authors have read and approved this manuscript for publication.
